# Human Tendon Stem Cells Better Maintain Their Stemness in Hypoxic Culture Conditions

**DOI:** 10.1371/journal.pone.0061424

**Published:** 2013-04-16

**Authors:** Jianying Zhang, James H.-C. Wang

**Affiliations:** MechanoBiology Laboratory, Departments of Orthopaedic Surgery, Bioengineering, Mechanical Engineering and Materials Science, and Physical Medicine and Rehabilitation, University of Pittsburgh, Pittsburgh, Pennsylvania, United States of America; Ohio State University, United States of America

## Abstract

Tissues and organs *in vivo* are under a hypoxic condition; that is, the oxygen tension is typically much lower than in ambient air. However, the effects of such a hypoxic condition on tendon stem cells, a recently identified tendon cell, remain incompletely defined. In cell culture experiments, we subjected human tendon stem cells (hTSCs) to a hypoxic condition with 5% O_2_, while subjecting control cells to a normaxic condition with 20% O_2_. We found that hTSCs at 5% O_2_ had significantly greater cell proliferation than those at 20% O_2_. Moreover, the expression of two stem cell marker genes, Nanog and Oct-4, was upregulated in the cells cultured in 5% O_2_. Finally, in cultures under 5% O_2_, more hTSCs expressed the stem cell markers nucleostemin, Oct-4, Nanog and SSEA-4. In an *in vivo* experiment, we found that when both cell groups were implanted with tendon-derived matrix, more tendon-like structures formed in the 5% O_2_ treated hTSCs than in 20% O_2_ treated hTSCs. Additionally, when both cell groups were implanted with Matrigel, the 5% O_2_ treated hTSCs showed more extensive formation of fatty, cartilage-like and bone-like tissues than the 20% O_2_ treated cells. Together, the findings of this study show that oxygen tension is a niche factor that regulates the stemness of hTSCs, and that less oxygen is better for maintaining hTSCs in culture and expanding them for cell therapy of tendon injuries.

## Introduction

Tendons connect muscles to bones to enable joint movement. As a result, they are subjected to large mechanical loads and hence are frequently injured. Full recovery of injured tendons requires a long, complex healing process, particularly in the case of complete tendon rupture when tendon retraction occurs. Moreover, healed tendons consist of scar tissue that has lower mechanical strength than normal tendon tissue. This mechanical weakness not only impairs normal tendon function and joint kinematics, but also predisposes patients to further tendon injury [1].

Restoring normal structure and function to injured tendons is challenging and a number of ways are being discovered to promote tendon regeneration after injury. Tissue engineering is one such approach that uses cells, scaffolds and growth factors to effectively repair or regenerate injured tendons more effectively. Cell therapy in particular, is important in tissue engineering to repair injured tendons or other tissues. For example, bone marrow mesenchymal stem cells (BMSCs) in conjugation with collagen gels, have been used to repair injured tendons [2] although these have resulted in ectopic bone formation in rabbit tendon injury models [3]. In addition, embryonic stem cells (ESCs) have also been used to repair injured tendons. However, ESCs implantation could result in teratoma formation, which occurs due to difficulty in controlling ESCs differentiation *in vivo* when compared to adult stem cells such as BMSCs. These and other studies clearly indicate that stem cells from non-tendinous tissues may not be optimal to restore the normal structure and function of injured tendons using cell therapy.

Implantation of autologous tenocytes, which are resident tendon cells responsible for the maintenance and repair of tendons has resulted only in a slight improvement in tendon quality [4]. A new type of recently discovered tendon cells called tendon stem cells (TSCs) have a great potential to repair injured tendons and have been identified in humans, rabbits, rats and mice [5]–[7]. Like adult stem cells, TSCs have the capacity for self-renewal, which enables them to make more stem cells by cell division and also possess multi-differentiation potential, which enables them to become specialized cell types. Under normal conditions, TSCs differentiate into tenocytes [6]. However, when implanted with engineered tendon matrix (ETM), TSCs form tendon-like tissues in nude rats [8]. Therefore, TSCs may be an ideal cell source for tissue engineering approaches that could effectively repair injured tendons.

To obtain sufficient numbers of cells for cell therapy of injured tendons, TSCs must be expanded in culture. However, under regular culture conditions that use 95% air and 5% CO_2_, TSCs tend to differentiate and consequently lose their stemness quickly. *In vivo,* tendons, which are collagen-rich structures with only a few blood vessels, have low oxygen levels when compared to vascular-rich organs and tissues such as the lungs, heart, liver and kidneys where the oxygen levels range from 10 to 13% [9]. However, the effects of low oxygen concentrations on TSCs have not been completely defined yet. In this study, we tested the hypothesis that under hypoxic conditions TSCs better maintain their stemness. Indeed, our findings show that low oxygen tension enhances the stemness of TSCs, which was characterized by quicker cell proliferation, higher expression of stem cell markers *in vitro* and more extensive formation of tendon-like and non-tendon-like tissues *in vivo*.

## Materials and Methods

### Ethics Statement

Normal human knee tissues were obtained within 24 hours of death of donors from the Gift of Hope Organ and Tissue Donor Network (Elmhurst, IL) with approval from the local ethics committee (Gift of Hope Organ and Tissue Donor Network). Written consent from the families was obtained and approved by the Gift of Hope Organ and Tissue donor Network. Tissue specimens were obtained for investigation only. The protocol to use human tendon tissues for subsequent cell culture and animal studies was approved by the University of Pittsburgh IRB.

This project did not involve human subjects, and the authors conducting research did not obtain data through intervention or interaction with individuals or obtain identifiable private information.

In addition, protocol for the use of rats for *in vivo* experimentation was approved by the University of Pittsburgh IACUC. All animal surgery was performed under general anesthesia and efforts were made to minimize suffering.

### Control of Hypoxic and Normoxic Culture Conditions

We used a dedicated tri-gas incubator (Thermo Scientific Heracell 150i, Thermo Scientific, and Pittsburgh, PA) to achieve hypoxic conditions (5% O_2_) in cell culture experiments. Concentration of oxygen in the incubator was precisely controlled by two gas controllers and an oxygen sensor. Nitrogen and carbon dioxide gases were supplied using a nitrogen gas controller (Thermo Scientific) connected to two nitrogen tanks and a carbon dioxide gas controller connected to two carbon dioxide tanks. The set up was such that the supply of gas automatically switched from the first to the second tank when the first tank was empty. To avoid air flow into the incubator during brief openings of the door the incubator was separated into three isolated chambers with each chamber closed by double doors. With these control devices in place, oxygen concentration in the incubator was maintained at a constant level of 5% during all cell culture experiments.

To maintain normoxic culture conditions (20% O_2_) a regular tissue culture incubator (Thermo Scientific) was used. About 20% O_2_ concentration was achieved inside the incubator by feeding 95% air and 5% carbon dioxide from tanks.

### Human TSC Culture

Human TSC (hTSCs) were obtained from the patellar tendons of six young adult donors aged 26 to 49 years following our previously published method [6].

### Cell Proliferation Experiment

hTSCs were seeded into 6-well culture plates at a density of 40,000 cells/well in 3 ml DMEM growth medium with 20% FBS and maintained in the tri-gas incubator to achieve a 5% O_2_ culture condition or the regular incubator to provide a 20% O_2_ culture condition. Replacement medium for the cells cultured in the tri-gas incubator was prepared by pre-conditioning the medium in the tri-gas incubator for at least 30 min before use. The medium was changed every two days under both hypoxic and normaxic culture conditions. Colony formation by hTSCs cultured in the two oxygen conditions was tested by staining with methyl violet. Cell proliferation was determined by counting cells on days 1, 2, 6 and 12 after seeding, as previously described [10].

### Stem Cell Marker Expression

To characterize the stemness of hTSCs in hypoxic and normaxic culture conditions, we determined differential expression of stem cell markers in hTSCs in both culture conditions. Cells were seeded into 12-well plates at a density of 20,000 cells/well with 1.5 ml medium and cultured either with 5 or 20% O_2_ for 3–5 days. Expression of four stem cell markers including nucleostemin (NS), octamer-binding transcription factor 4 (Oct-4), Nanog and stage-specific embryonic antigen-4 (SSEA-4) was measured using immunocytochemistry. Briefly, hTSCs were fixed in 4% paraformaldehyde in PBS for 20 min at room temperature. For Oct-4, Nanog and nucleostemin staining fixed cells were treated with 0.5% Triton-X-100 in PBS for 15 min and washed with 2% mouse or goat serum-PBS for 30 min. The cells were then incubated with either mouse anti-human Oct-4 (1∶500), rabbit anti-human Nanog (1∶500) or goat anti-human nucleostemin (1∶500) overnight at 4°C. After washing in PBS three times, the cells were again incubated with either Cy-3-conjugated goat anti-mouse IgG antibodies (1∶1000), Cy3-conjugated goat anti-rabbit IgG (1∶500) or Cy-3-conjugated donkey anti-goat IgG antibodies (1∶500) for 2 hrs at room temperature to detect Oct-4, Nanog and nucleostemin respectively. To stain for SSEA-4, fixed cells were blocked with 2% mouse serum for 1 hr and incubated with mouse anti-human SSEA-4 antibody (1∶500) for 2 hrs at room temperature. After subsequent washing with PBS, TSCs were treated with Cy3-conjugated goat anti-mouse IgG antibody (1∶1000) for 1 hr at room temperature. Stained cells were then examined using fluorescence microscopy. All antibodies were obtained from Chemicon International (Temecula, CA), BD Biosciences (Franklin Lakes, NJ), Neuromics (Edina, MN) or Santa Cruz Biotechnology Inc. (Santa Cruz, CA).

### Multi-differentiation Potentials

The differentiation capacity of hTSCs in hypoxic and normaxic culture conditions was examined *in vitro* by testing their abilities to undergo adipogenesis, chondrogenesis and osteogenesis. Cells at passage 1 were seeded into 6-well plates at a density of 24 × 10^4^ cells/well in basic growth medium (DMEM plus 10% FBS) and cultured in either 5 or 20% O_2_. To measure adipogenic potential, hTSCs were cultured in adipogenic induction medium (Millipore, Billerica, MA) that consists of basic growth medium supplemented with dexamethasone (1 µM), insulin (10 µg/ml), indomethacin (100 µM) and isobutylmethylxanthine (0.5 mM). To determine chondrogenic potential, hTSCs were cultured in basic growth medium supplemented with proline (40 µg/ml), dexamethasone (39 ng/ml), TGF-β3 (10 ng/ml), ascorbic 2-phosphate (50 µg/ml), sodium pyruvate (100 µg/ml) and insulin-transferrin-selenious acid mix (50 mg/ml) (BD Bioscience, Bedford, MA). Finally, osteogenic potential of hTSCs in both hypoxic and normaxic culture conditions was studied by culturing cells in osteogenic induction medium (Millipore, Billerica, MA) consisting of basic growth medium supplemented with dexamethasone (0.1 µM), ascorbic 2-phosphate (0.2 mM) and glycerol 2-phosphate (10 mM). hTSCs were grown in above three media for 21 days followed by Oil red O assay for adipogenesis, Safranin O assay for chondrogenesis and Alizarin red S assay for osteogenesis as described previously [6].

### Semi-quantification of the Extent of hTSC Differentiation

For the semi-quantification of cell differentiation, twelve images of each well were randomly taken under a microscope (Nikon eclipse, TE2000-U). Then areas with positive staining were manually identified from each picture and computed by SPOT™ imaging software (Diagnostic Instruments, Inc., Sterling Heights, MI). Proportion of positive staining was calculated by dividing the positively stained area by the total area viewed under the microscope. These values were obtained for all twelve images of a well and their average was used to represent the percentage of positive staining, which is the extent of cell differentiation in the respective induction media described under multi-differentiation potentials.

### Quantitative Real-time PCR (qRT-PCR)

To measure the stemness of hTSCs under hypoxic (5% O_2_) and normaxic culture conditions (20% O_2_) we performed qRT-PCR analysis. Total RNA was extracted from hTSCs using an RNeasy Mini Kit with an on-column DNase I digest (Qiagen). First-strand cDNA was synthesized by reverse transcribing 1 µg total RNA with SuperScript II (Invitrogen) in a 20 µl reaction volume. The conditions for cDNA synthesis included: 65°C for 5 min followed by cooling at 4°C for 1 min, then 42°C for 50 min and finally 72°C for 15 min. qRT-PCR was carried out using 2 µl cDNA (approximately 100 ng RNA) in a 25 µl PCR reaction volume using QIAGEN QuantiTect SYBR Green PCR Kit (Qiagen) in a Chromo 4 Detector (MJ Research). To determine stemness of TSCs gene-specific primers of human Oct-4, Nanog, and tenocyte-related genes, including collagen type I and tenascin C were used. Glyceraldehyde-3-phosphate dehydrogenase (GAPDH) was used as an internal control. Forward and reverse primers for all genes were designed based on previously published sequences [11]–[13] and were synthesized by Invitrogen (Carlsbad, CA). Relative gene expression levels in hTSCs under hypoxic and normaxic culture conditions were determined using the formula ΔΔCT = (CT_target_−CT_GAPDH_)_Hypoxia_−(CT_target_−CT_GAPDH_)_Normoxia_, where CT represents cycle threshold of each RNA sample. At least three replicates were performed for each gene and each experimental condition.

### Preparation of hTSCs for *in vivo* Implantation

hTSCs for implantation were prepared by plating cells from passage 2 into two 24-well plates at a seeding density of 6×10^4^/well and were allowed to grow in 5 or 20% O_2_ culture conditions. After one week, cells under both culture conditions were collected and each was mixed separately with 0.5 ml 5% ETM made from rabbit patellar tendon samples according to our previously published method [8] or 0.5 ml Matrigel (Cat. # 354234, BD Biosciences, Bedford, MA). The cell-ETM or cell-Matrigel composites were then reseeded into a 24-well plate and cultured overnight with 5 or 20% O_2_ to maintain hypoxic or normaxic conditions respectively.

### *In vivo* Implantation Experiment

Four 10 weeks old female nude rats weighing between 200–250 g were used for hTSC implantation experiments. Protocol for the use of rats for *in vivo* experimentation was approved by the University of Pittsburgh IACUC. Before implantation, all rats were given general anesthesia by intramuscular injection of a mixture of ketamine hydrochloride (75 mg/kg body weight) and xylazine hydrochloride (5 mg/kg body weight). Two rats each were implanted with hTSCs cultured in 5 or 20% O_2_ conditions. A total of six distinct wounds were made on the back of each rat and each wound was filled with a piece of cell-ETM or cell-Matrigel composite. Three weeks after implantation, the wound sites were opened and tissues in the area were harvested. The tissue samples were then immersed in frozen section medium (Neg 50; Richard-Allan Scientific; Kalamazoo, MI) in pre-labeled base molds and were quickly frozen in 2-methylbutane chilled with liquid nitrogen. Frozen tissue blocks were then placed on dry ice and stored in -80°C until further use for histological and immunohistochemical analyses. At least three replicates were performed for each experimental condition.

### Detection of hTSC Differentiation *in vivo*

Frozen tissue blocks were cut into 8 µm thick sections, fixed in 4% paraformaldehyde for 15 min and stained with mouse anti-human collagen type I (1∶100, Millipore, Cat. #MAB1340; Temecula, CA), mouse anti-human adiponectin (1∶300, Millipore; Cat. #MAB3604; Temecula, CA), mouse anti-human collagen type II (1∶100, Millipore, Cat. #MAB1330, Temecula, CA) and mouse anti-human osteocalcin (1∶200, Abcam, Cat #13418, Cambridge, MA) at room temperature for 2 hrs. Cy-3 conjugated goat anti-mouse IgG (1∶500, Jackson ImmunoResearch Laboratories, Inc., Cat. #115-165-146, West Grove, PA) was used as the secondary antibody to detect collagen type I, collagen type II and osteocalcin at room temperature for 2 hrs. FITC-conjugated goat anti-mouse IgM (1∶500, Santa Cruz Biotechnology, Cat. #sc-2082, Santa Cruz, CA) was used as the secondary antibody to detect adiponectin. The tissue sections were also treated with Hoechst 33342 (Sigma, Cat. #B2261, St. Louis, MO) to stain nuclei.

### Statistical Analysis

One-way analysis of variance (ANOVA) followed by either Fisher’s predicted least-square difference (PLSD) for multiple comparisons or two tailed, paired or unpaired student *t*-test were performed wherever applicable. Differences between two groups (hypoxic vs. normaxic conditions) were considered significant when P-value was below 0.05.

## Results

**Figure 1:**
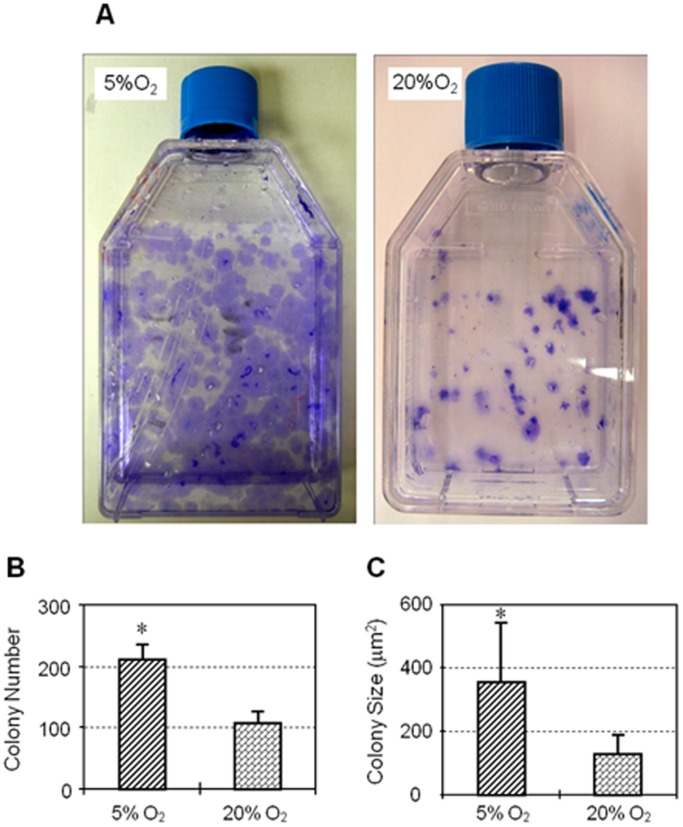
A. Colony formation by hTSCs under hypoxic and normaxic culture conditions. T-25 flask maintained in 5% O_2_ (left) shows a number of large methyl violet stained hTSC colonies while the flask in 20% O_2_ (right) has fewer and smaller colonies. B. Quantification of hTSC colony numbers under hypoxic and normaxic conditions. Colony numbers in 5% O_2_ were twice that in 20% O_2_. Two flasks of hTSCs each were used to calculate colony numbers in hypoxic and normaxic conditions. C. Quantification of hTSC colony sizes under hypoxic and normaxic conditions. Colony size of hTSCs in hypoxic condition (5% O_2_) was about 2.8 times larger than in normaxic condition (20% O_2_). Asterisks represent P<0.05.

**Figure 2:**
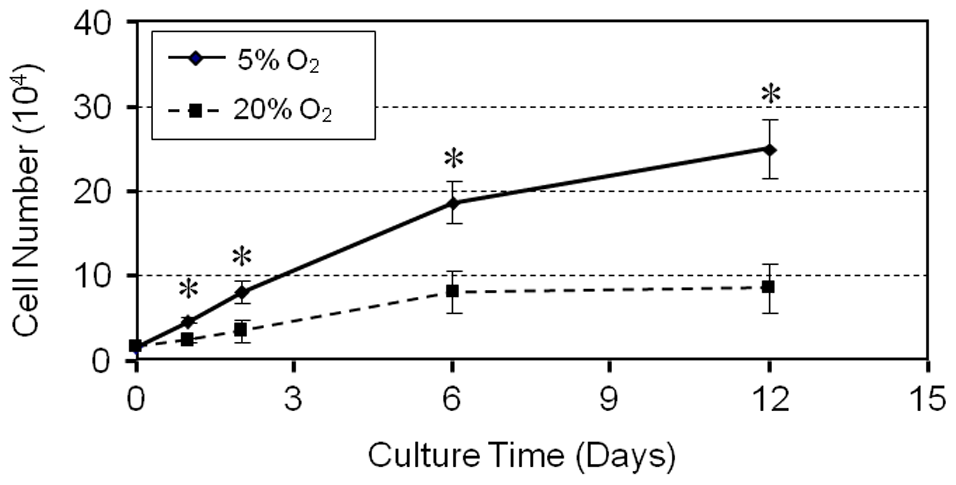
Proliferation of hTSCs cultured under hypoxic and normaxic culture conditions. hTSCs were grown in DMEM growth medium with FBS under hypoxic or normaxic conditions and colony formation was determined by counting cells stained with methyl violet. While the cells grew at both culture conditions, at all time points (days 1, 2, 6, and 12), hTSCs at 5% O_2_ grew significantly quicker than at 20% O_2_.

To determine the effects of hypoxia, similar numbers of hTSCs were seeded and cultured in both hypoxic and normaxic conditions. We found that hTSCs cultured in 5% O_2_ formed more colonies that were also larger than cells cultured in 20% O_2_ (Figure 1A, 1B). Colonies formed in 5% O_2_ were twice as many as those in 20% O_2_ (Figure 1B) and the colony size in 5% O_2_ was on an average about 2.8 times larger than those in 20% O_2_ (Figure 1C). In addition, proliferation of hTSCs in both culture conditions increased in the days following seeding and the number of cells in 5% O_2_ was higher than in 20% O_2_ from day 1 through days 2, 6 and 12 (Figure 2).

**Figure 3:**
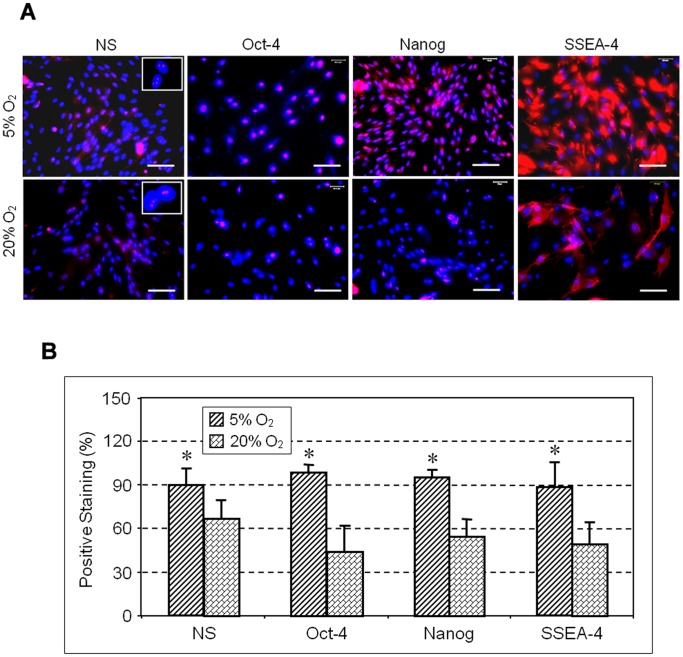
A. The expression of stem cell markers by hTSCs under hypoxic and normaxic culture conditions. hTSCs grown under hypoxic or normaxic conditions were analyzed by immunocytochemistry using specific antibodies to determine stem cell marker expression (See materials and methods for details). Compared to normaxic condition (20% O_2_), more hTSCs at hypoxic condition (5% O_2_) expressed nucleostemin (NS), Oct-4, Nanog, and SSEA-4, all of which are known stem cell markers. Insets indicate NC proteins in the nuclei of hTSCs. Nuclei were stained with Hoechst 33342. Scale bars: 100 µm. B. Semi-quantification of stem cell markers by staining. hTSCs specifically stained for NS, Oct-4, Nanog and SSEA-4 by immunocytochemical staining were counted to calculate percentage staining. As indicated, significantly higher percentages of hTSCs cultured under 5% O_2_ conditions expressed the stem cell markers (NS, Oct-4, Nanog, and SSEA-4) compared to those cultured under 20% O_2_ conditions (*P<0.05, with respect to hTSCs under 20% O_2_ culture conditions). Scale bars: 100 µm.

**Figure 4:**
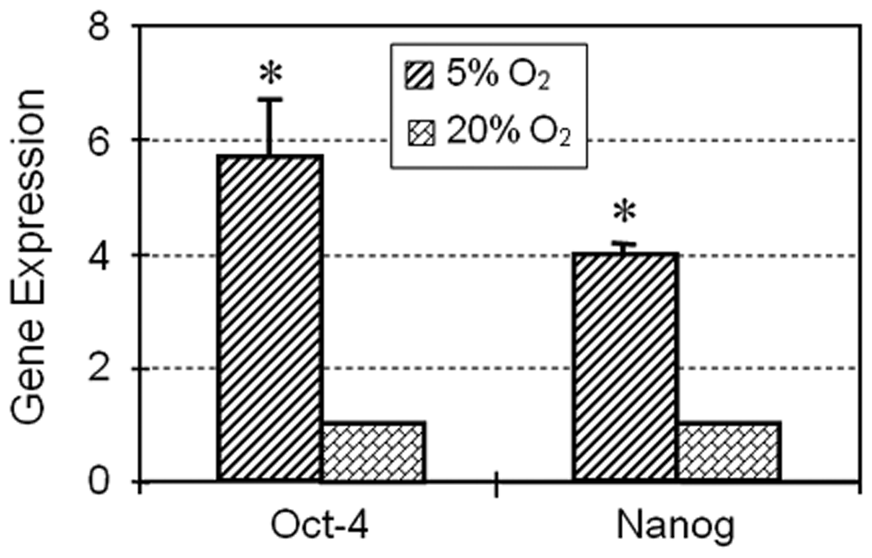
Stem cell gene analysis by qRT-PCR. Total RNA extracted from hTSCs grown under hypoxic or normaxic conditions was used to synthesize cDNA, which was used as a template in qRT-PCR using primers specific to Oct-4 and Nanog. GAPDH was used as an internal control. Y- axis represents relative gene expression when compared to GAPDH expression levels. Ct values were normalized against hTSCs cultured under 20% O_2_. Both stem cell marker genes (Oct-4 and Nanog) cultured at 5% O_2_ culture conditions were expressed at significantly higher g005levels than those cultured at 20% O_2_ culture conditions.

**Figure 5:**
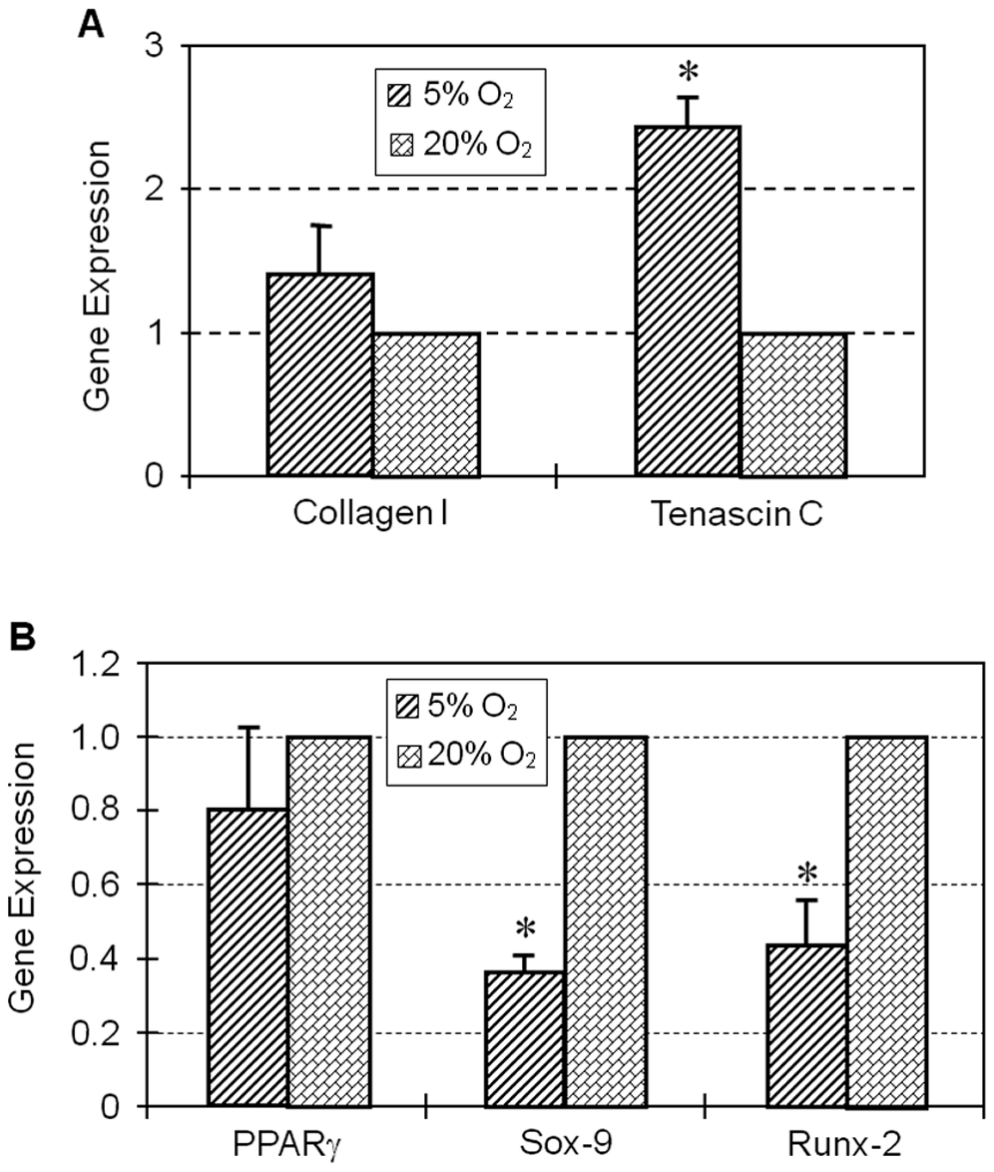
A. Tenocyte related gene expression by hTSCs under hypoxic and normaxic culture conditions. Total RNA extracted from hTSCs grown under hypoxic or normaxic conditions was used to synthesize cDNA, which was used as a template in qRT-PCR using primers specific to Collagen-1 and Tenascin C. GAPDH was used as an internal control. Y- axis represents relative gene expression when compared to GAPDH expression levels. Ct values were normalized against hTSCs cultured under 20% O_2_. At both oxygen conditions (5% and 20% O_2_), there was no significant difference in the expression of collagen type I, but the expression of tenascin C in the hypoxic group was significantly higher than in the normaxic group (*P<0.05). B. Non-tenocyte related gene expression by hTSCs under the above two oxygen conditions. Total RNA extracted from hTSCs grown under hypoxic or normaxic conditions was used to synthesize cDNA, which was used as a template in qRT-PCR using primers specific to PPARγ, Sox-9 and Runx-2. GAPDH was used as an internal control. Y- axis represents relative gene expression when compared to GAPDH expression. Ct values were normalized against hTSCs cultured under 20% O_2_. The cellular expression of PPARγ, a marker for adipogenesis, was not significantly different in 5 and 20% O_2_ conditions. However, Sox-9 and Runx-2 (markers for chondrogenesis and osteogenesis, respectively) were expressed at significantly lower levels when hTSCs were cultured at 5% O_2_ condition in comparison to 20% O_2_ (*P<0.05, respective to hTSCs that were under normaxic conditions).

Expression of stem cell markers NS, Oct-4, Nanog and SSEA-4 determined by immunocytochemistry was also higher in colonies cultured in 5% O_2_ compared to hTSCs grown in 20% O_2_ (Figure 3A). Semi-quantification of the immuno-stained cells further showed that more than 90% of hTSCs cultured in 5% O_2_ were NS positive compared to 66% in hTSCs cultured in 20% O_2_. Oct-4 expression was also higher (98%) in hTSCs cultured at 5% O_2_ when compare to cells cultured in 20% O_2_ (44%). Similarly, the expression of Nanog and SSEA-4 in hTSCs cultured in 5% O_2_ was 95 and 88% respectively when compared to the lower percentages (55 and 49%) observed in hTSCs cultured in 20% O_2_ (Figure 3B). Consistent with these results RT-PCR analysis also showed higher expression levels of both Oct-4 and Nanog genes in 5% O_2_ compared to 20% O_2_ (Figure 4). Cells cultured in both conditions showed no significant difference in the expression of the tenocyte-related gene, collagen type I, but the level of tenascin C expression was more than 2-fold higher in 5% O_2_ compared to 20% O_2_ (Figure 5A). Moreover, the expressions of non-tenocyte-related genes Sox-9 and Runx-2 were significantly lower in 5% O_2_ than in 20% O_2_ culture conditions while expression of PPARγ was marginally lower (Figure 5B).

**Figure 6:**
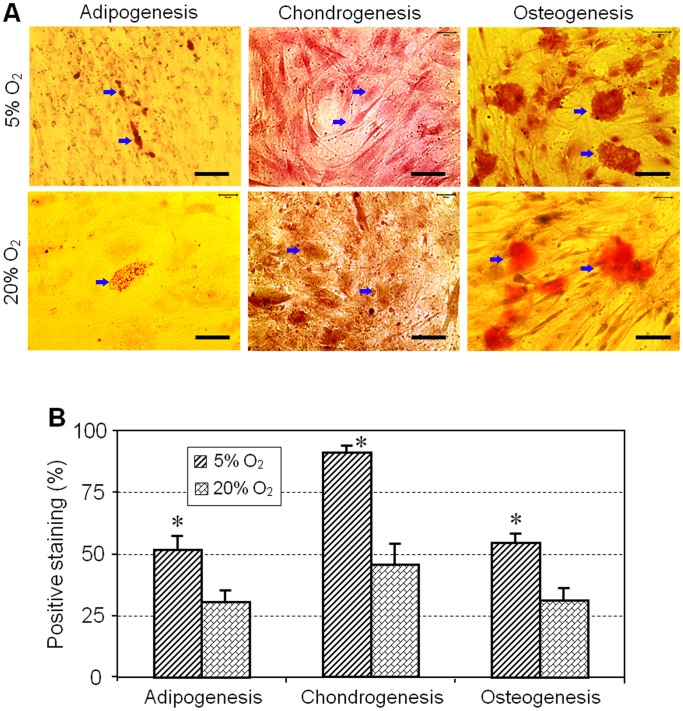
A. Multi-differentiation capacity of hTSCs under hypoxic and normaxic culture conditions hTSCs were separately grown under both hypoxic or normaxic conditions in adipogenic, chondrogenic and osteogenic induction media for 21 days followed by staining with Oil red O for adipogenesis, Safranin O for chondrogenesis and Alizarin red S for osteogenesis. It is apparent that compared to hTSCs at 20% O_2_ condition, cells grown at 5% O_2_ culture condition formed more extensive lipids, proteoglycan accumulation, and calcium deposition, as revealed by Oil red O assay, Safranin O assay, and Alizarin red S assay, respectively. Positively stained cells are indicated by arrows. Scale bars: 100 µm. B. Semi-quantification of the staining results by three assays. Positively stained cells were counted to calculate percentage staining. More hTSCs at 5% O_2_ condition were found to differentiate into adipocytes, chondrocytes and osteocytes than hTSCs at 20% O_2_ condition (*P<0.05, with respect to hTSCs that were under normaxic condition).

We next examined the multi-differentiation capacity of TSCs under hypoxic and normaxic culture conditions. After 21 days in culture, the degree of adipogenesis, chondrogenesis and osteogenesis of hTSCs was more extensive in 5% O_2_ condition compared to 20% O_2_ (Figure 6A). Semi-quantitative analysis was also consistent with these results with the percentages of hTSCs that differentiated into adipocytes, osteocytes and chondrocytes about 51, 90 and 54% respectively in 5% O_2_ conditions when compared to lesser percentages of 31, 46 and 31% respectively in 20% O_2_ (Figure 6B).

**Figure 7:**
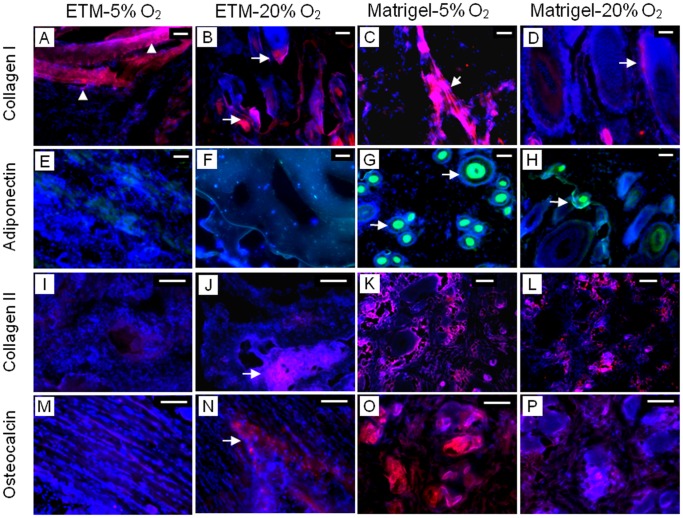
In vivo implantation results of hTSCs after culture in hypoxic and normaxic conditions. hTSCs grown under both hypoxic or normaxic conditions were implanted into nude rats. Implantation of the cells embedded in ETM (ETM-5% O_2_) resulted in the formation of tendon-like structures (A, triangles) compared to only spotty areas stained with human collagen type I (B, arrows) when hTSCs treated with 20% O_2_ and embedded in the same ETM (ETM-20% O_2_) were implanted *in vivo*. In addition, implantation of ETM-5% O_2_ hTSCs led to little formation of adiponectin (E), collagen type II (I), and osteocalcin (M); in contrast, implantation of ETM-20% O_2_ hTSCs exhibited strong staining for collagen type II (J, arrow) and osteocalcin (N, arrow). When hTSCs were treated with 5% O_2_ and embedded in Matrigel, implantation of the cell-Matrigel composites formed more extensive tendinous (C, D, arrows) and non-tendinous tissues (G, green; K, red; O, red) compared to hTSCs treated with 20% O_2_ and embedded in Matrigel (H, green; L, red; P, red). Red represents collagen type I (A–D); Green represents adiponectin (E–H); Red represents collagen type II (I–L) and red represents osteocalcin (M–P). In all figures blue represents nuclei stained with Hoechst 33342. Scale bars: 100 µm.G.

To further characterize hTSCs after exposure to hypoxic and normaxic conditions, we implanted the cells grown under the two conditions into nude rats subcutaneously. Three weeks after implantation hTSCs cultured in 5% O_2_ and embedded in ETM resulted in extensive formation of bands that corresponded to tendon-like structures, as evidenced by strong staining for human collagen type I (hCT-I). In contrast, hTSCs cultured in 20% O_2_ before embedding in ETM and transplantation, showed only discreet areas which were stained positive for hCT-I. Staining for adiponectin (a marker for adipogenesis), collagen type II (a marker for chondrogenesis) and osteocalcin (a marker for osteogenesis) showed minimal formation of fatty, cartilage, and bony tissues in 5% O_2_ treated hTSCs and embedded in ETM (ETM-5% O_2_) whereas the cells treated with 20% O_2_ and embedded in ETM (ETM-20% O_2_) formed well- developed cartilage and bony tissues. Similarly, when 5 and 20% O_2_ treated hTSCs were embedded in Matrigel and implanted, we observed formation of all four types of tissues (tendinous, fatty, cartilage-like and bony tissues), which was more extensive in 5% O_2_ treated hTSCs compared to 20% O_2_ treated hTSCs (Figure 7). Semi-quantitative analysis further showed that compared to normaxic condition, hypoxic condition resulted in higher amounts of both tendinous protein (collagen type I) and non-tendinous proteins (adiponectin, collagen type II and osteocalcin) (data not shown).

## Discussion

Tendon injury is common in both occupational and athletic settings. Currently, there are no effective means to restore normal structure and function to injured tendons. TSCs, which were only recently identified, are tendon-specific adult stem cells that are thought to play a critical role in the repair of injured tendons. Therefore, TSCs may be an optimal cell source for effective tissue engineering of injured tendons. However, the major obstacle using such cell therapy is that once TSCs are isolated from tendons and grown in a conventional *in vitro* environment, they tend to differentiate quickly. Considering that TSCs *in vivo* are under hypoxic conditions due to poor vascularity in tendon substances, we designed this study to investigate the effects of hypoxic conditions on hTSCs. By performing cell culture experiments, we have shown that the stemness of hTSCs cultured in 5% O_2_ was better than hTSCs at 20% oxygen levels. Specifically, under the hypoxic culture condition (5% O_2_), growth of hTSCs was faster, higher number of cells expressed stem cell markers (NS, Oct-4, Nanog and SSEA-4) and the expression level of stem cell marker genes (Oct-4 and Nanog) was also significantly higher. In addition, while tenocyte-related gene expression levels were similar under both hypoxic and normaxic conditions (5% O_2_ vs. 20% O_2_), non-tenocyte related gene expression at hypoxic condition was significantly lower than in normaxic condition. Moreover, hTSCs cultured under the hypoxic condition (5% O_2_) exhibited more potent multi-differentiation capacity in terms of adipogenesis, chondrogenesis and osteogenesis. By performing an *in vivo* implantation experiment, we were also able to show that when implanted together with ETM, hTSCs in hypoxic condition produced more extensive tendon-like tissues than in normaxic condition. The hypoxic condition also resulted in more tendinous and non-tendinous tissues than normaxic condition when both were implanted with Matrigel. The *in vivo* results further showed that hypoxic condition enhanced multi-differentiation potential of hTSCs.

The findings of this study show that oxygen is an important niche factor for the maintenance of stemness by hTSCs. These findings also indicate that for effective tissue engineering of injured tendons, TSCs should be cultured in a hypoxic environment. This hypoxic condition can promote TSCs’ self-renewal, thus allowing sufficient numbers of TSCs to be obtained for tissue engineering, which may repair injured tendons more effectively. The high self-renewal rate of hTSCs under a hypoxic condition is consistent with the concept that adult stem cells like hTSCs ensure maintenance of their pool for tissue repair or regeneration when the tissue is injured [14]. Finally, this study also indicates that caution should be exercised before using the so-called hyperbaric oxygen therapy to treat injured tendons, at levels as high as >20% O_2_ that could potentially deplete the TSCs pool quickly by promoting their differentiation into specialized cell types and consequently could hinder the repair of tendons after re-injury.

Many studies have investigated the effects of various hypoxic conditions on cells. For example, compared to normaxic condition (20% O_2_), hypoxic condition (1.5% to 5% O_2_) increases proliferation of human mesenchymal stem cells (hMSCs) [15]. In addition, hMSCs grown in 2% O_2_ exhibited enhanced colony-forming capabilities and had a higher expression of Oct-4 [16], [17]. Hypoxic conditions also produced greater numbers of stem cell colonies that proliferated more rapidly in culture. Rat MSCs cultured in 5% O_2_ produced more bone than cells cultured in 20% O_2_ when the cells were loaded into porous ceramic cubes and implanted into animals [18]. In addition, hESCs in 20% O_2_ culture condition showed decreased cell proliferation and reduced expression of Nanog and Oct-4 genes, and Oct-4 protein, compared to 5% O_2_ culture condition [19]. Our results were consistent with the findings of these previous studies.

However, while our study found that the hypoxic condition at 5% O_2_ enhanced differentiation potential of hTSCs, a previous study showed a decrease in the multi-differentiation potential of hTSCs under hypoxic condition (2% O_2_) [20]. There are several possible reasons for this discrepancy. First, our study used a tri-gas incubator, whereas their study used a hypoxic chamber that controlled oxygen levels in a regular incubator with 20% O_2_. The two different means of controlling oxygen concentrations could result in huge differences in the conditions under which hTSCs were cultured; i.e., nearly constant oxygen levels vs. fluctuating oxygen levels during culture experiments. Second, the initial states of hTSCs in both studies could have been different. For example, Oct-4 expressing hTSCs vs. tendon progenitor cells that do not express Oct-4 that consequently resulted in differences in cellular responses to similar hypoxic conditions. Finally, there could be differences in experimental conditions used in hTSCs culture including the density of cells, depth of medium, pre-conditioning of medium and cellular respiration, all of which could alter the oxygen tension at the surface of cultured cells, consequently leading to differential responses of hTSCs to hypoxic conditions.

Because there are no specific stem cell markers for hTSCs, we used general stem cell markers (NS, Oct-4, Nanog, and SSEA-4) to characterize their stemness under both hypoxic and normaxic conditions. Nucleostemin (NS), that controls cell cycle progression, is exclusively expressed in stem cells, and is therefore not expressed in committed and terminally differentiated cells [21]. Nanog, a unique homeobox transcription factor, was reported to be expressed in pluripotent stem cells, and its expression was associated with stem cell differentiation [22]. Typically expressed in embryonic stem cells (ESCs) during development, Oct-4 is a transcription factor that is known to mediate pluripotency in ESCs [23]. Oct-4 is also essential for maintaining pluripotent stem cells, and is not expressed in differentiated cells [24]. Finally, SSEA-4 is a transcription factor specific to undifferentiated pluripotent human or mouse stem cells [25]–[27]. Thus, the higher expression levels of these stem cell markers in hypoxic condition (5% O_2_) observed in this study indicate that more hTSCs were kept in an undifferentiated state and self-renewed when they were cultured at hypoxic condition (5% O_2_) than at normaxic condition (20% O_2_).

It is generally accepted that 3 to 5% oxygen levels are present in tissues, although the actual O_2_ concentration *in situ* depends on vascularization of the tissue and its metabolic activity [15]. To our best knowledge, the physiological oxygen tension of the human patellar tendon remains unknown. In the articular cartilage, however, oxygen tension is known to be less than 10% at the surface and less than 1% in the deepest layer [28]. Considering that tendons are largely avascular, it is likely that their oxygen tension is higher than 1% but lower than 10%. This is the reason we chose a 5% O_2_ level in this study. Use of 5% O_2_ level also makes it possible to control oxygen levels in an incubator more precisely, as too low levels of oxygen, which creates a high gradient of oxygen against the environment, is technically demanding in terms of precisely controlling constant oxygen levels to culture cells.

There are a few limitations associated with this study. First, we grew hTSCs in plastic dishes, which itself is “foreign” to hTSCs and therefore may cause cell differentiation in culture. Our previous study showed that TSCs grown on tendon matrix coated plastic surfaces can encourage self-renewal of TSCs. Therefore, it seems reasonable to speculate that culture of hTSCs in tendon matrix under a hypoxic condition will result in an even higher stemness of hTSCs, especially in long term cell culture. Second, tendons *in vivo* are constantly subjected to mechanical loading because of their role in the transmission of muscular forces to bones. Mechanical loading, however, was not included in our cell culture experiment although, our previous study showed that mechanical loading itself can regulate TSC functions including proliferation and differentiation [29]. Therefore, future studies should investigate the combined effects of hypoxic conditions and mechanical loading on TSCs. Finally, the molecular mechanisms that are responsible for enhanced stemness in hTSCs as shown in this study are yet to be determined. Nevertheless, it is known that when cells sense changes in oxygen availability, they initiate survival responses by inducing and increasing the expression of hypoxic inducible factor (HIF) in hMSCs [15]. HIF-2α, an isoform of HIF, was also reported to regulate hESC pluripotency and proliferation under hypoxic conditions [19]. Therefore, it is possible that HIF also regulates hypoxic responses of hTSCs. This possibility is supported by the previous finding that HIF regulates pluripotency and proliferation in hESCs cultured at hypoxic culture conditions [19] by activating the expression of Oct-4, which is known to control the self-renewal and multi-potency of stem cells [30].

In conclusion, using *in vitro* and *in vivo* experimental approaches, we have shown that culture condition using low oxygen level of 5% encourages self-renewal of hTSCs and, as a result, yields more abundant hTSCs than the conventionally used culture condition at 20% oxygen level. Higher number of hTSCs in 5% oxygen conditions will enable the use of these tendon specific stem cells for cell therapy of injured tendons. Future studies are required to investigate the combined effects of low-oxygen culture conditions with other TSC’s niche factors, including tendon matrix and mechanical loading, on TSC functions.
